# A Case of Natrophagie

**Published:** 1858-08

**Authors:** 

**Affiliations:** Crown Physician, in Bonn.


ARTICLE VI.

(Translated by DR. BYFORD, from the *All. Med. Cent. Zeitung.*)

### A CASE OF NATROPHAGIE.

RELATED BY DR. BÖCKER, CROWN PHYSICIAN, IN BONN.

Mr. Titz, formerly an apothecary, now a stock-jobber, sixty-two years of age, enjoyed excellent health during early life, and up to 1840. About this time, he became subject to daily emesis, which lasted to 1842, at which time he had become very much emaciated and bedridden. He now underwent treatment at Hamburg, with very great relief; but it was not long until the vomiting returned to its former distressing extent. The substance ejected was dark mucous coffee-grounds looking stuff, sometimes decidedly bloody, and very sour. The emaciation now became very great indeed.

Mr. Titz, from the fall of 1842 to the beginning of 1843, took daily a half ounce of bi-carbonate of soda, and from the year 1843 to 1854, daily, one whole ounce of the same substance, with complete relief to the vomiting. He sometimes took the salt with a little water, but ordinarily he chewed or swallowed the dry crystal. The taste became quite tolerable, in fact agreeable. He took the ounce in three portions, either shortly before or immediately after eating. It was for a quarter of an hour followed by eructations of carbonic acid gas.

In a few days after beginning, in 1842, to take the bi-carbonate, all the symptoms began to improve. The vomiting and acid eructations ceased, the appetite returned, the digestion became good, defecation regular and fæces natural in appearance, the patient assumed the entire appearance of good health, and in a few months complete health was restored. Jocundity and rotundity took the place of his former meagreness and melancholy.

In 1854, Mr. Titz stopped the use of the soda; soon he felt weight in the epigastrium, and experienced flatulency, acid eructations, somnolence, weakness, yawning, asthma and rheumatic pains. He was for several weeks treated by different physicians, most of whom attributed his symptoms to the inordinate use of soda. He grew worse, until, in despair, he resumed the bi-carbonate of soda, from six drachms to an ounce daily. From this time forward he improved, until, in a short time, every symptom had vanished, and up to this time Mr. Titz has enjoyed uninterrupted good health.

When he goes on a journey, and for a few days neglects his soda, the symptoms begin to make their appearance, to be entirely dissipated by a return to the remedy in full doses. It is necessary that at least six drachms or an ounce be taken daily to have the proper effect. His habits now are very regular. He rises at seven o'clock, takes his bread and coffee, dines at one o'clock, with a good appetite, taking afterward a glass of wine or beer, sleeps an hour, goes out walking, in the afternoon drinks a cup of coffee, mingles in society of evenings, takes a glass of wine or beer, sups, goes to bed at ten o'clock evening, and sleeps soundly till morning.

Mr. Titz is robust and healthy, of blooming countenance, strong in muscle and corpulent. His digestion, notwithstanding the extraordinary use of his soda, is good; his bowels are regular; the urine is alkaline, clear wine color and without sediment, ferments upon the addition of acid, and contains a great deal of bi-carbonate of soda.*

The lungs are perfectly sound. There seems to be some deficiency in the left auriculo-ventricular valves of the heart, which, however, causes no embarrassment. Pulse, sixty-five to the minute. The liver, spleen, kidneys, none of the abdominal organs, give any sign of disease whatever. In fact, Mr. Titz, for a person of his age, is a model of unexceptionable health. His four children, all grown, are also healthy. If, in consequence of an omission of his accustomed wine or beer, his appetite becomes poor, a somewhat large dose of the soda sets all things right with him in a few hours.

This brief history of a very singular case possesses several points of interest. We may from it, I think, draw a useful lesson, and have our views enlarged in regard to physiological processes.

---

* It would have been interesting to ascertain whether the fæces did not contain the bi-carbonate of soda, but Mr. Titz would not give me an opportunity to experiment on them.

If it were true that alkaline mineral waters are a cure for obesity, that alkalies destroy the fat, Mr. Titz should be very much emaciated, instead of being so fat and lusty.

It is said the long use of alkalies deprives the bones of chalk. If this were true, Mr. Titz should have been cartilaginous before this time; but we find, that after fifteen years' use of soda, his osseous system is strong and firm. Also, if it is necessary for the gastric juice to be acid for complete digestion, what an enormous quantity of acid must the stomach of Mr. Titz secrete.

I should have been very glad to have entered into a series of research with Mr. Titz, in order to ascertain the effects of soda upon the organism, more particularly the secretions, but he would run no farther risk of effects which the withdrawal of the soda from him seemed invariably to produce; and to ascertain its precise influence, it was indispensable to withdraw it for some time. An analysis of the blood would also have been exceedingly interesting to me, but it was impossible to procure any for the purpose.

Although it was not in my power to develop this case in all its important aspects, I hope the foregoing account of it may not be altogether profitless.

Bonn, June 2d, 1858.

